# Risk Management Knowledges about Oysters for Raw Consumption and Norovirus

**DOI:** 10.14252/foodsafetyfscj.D-20-00014

**Published:** 2020-09-25

**Authors:** Kazuo Koyama, Azusa Hirakawa, Chie Uehara, Itsuko Horiguchi

**Affiliations:** 1 Food Safety Commission Secretariat, Cabinet Office, Government of Japan, Akasaka 5-2-20, Minato-ku, Tokyo 107-6122, Japan; 2 The Support Center for Clinical Pharmacy Education and Research, Tokyo University of Science, 1-3 Kagurazaka, Shinjuku-ku, Tokyo 162-8601, Japan

**Keywords:** food safety, norovirus, oyster, poisoning, questionnaire, risk communication

## Abstract

This study used questionnaires to evaluate knowledge levels of risk management of raw-consumption of oysters and of norovirus as health hazards among monitors signed up for Food Safety Commission of Japan (FSCJ) having work experiences in food fields. The mean scores of monitors on norovirus knowledge were relatively high (79%), but on oyster raw-consumption were low (64%). Scores varied depending on occupational experiences; highest among administrative officials, high among researchers in food companies, and low among medical workers and educators. The higher scores with more practical experiences for risk management of oyster raw-consumption and norovirus were observed among the monitors. These monitors were expected better to recognize the risks, wheres only few monitors among the opinion-leaders replied correctly to all the questions. These results suggest the need of improvement on the management system for oyster raw-consumption, from the current complicated to the more precise and reinforced for consumers. To efficiently manage the risk associated with the consumption of raw oysters, the government should provide more relevant information of risk management to persons having interest, particularly influencers, in order to disseminate information and to improve knowledge among cooks and consumers.

## 1. Introduction

Based on the official records of Japan in 2018, 17,282 patients (1,330 incidents) were reported with food poisoning^1^^)^. Norovirus was the most common cause of severe food poisoning sharing 8,475 patients (256 incidents), accounting for 49% of all patients reported. Thus, norovirus affected 33 people per incident in average. 

Norovirus causing gastroenteritis is contracted through contact or droplet infection due to the shedding of the virus in the vomit or feces of infected individuals^2^^,^^3^^,^^4^^)^. Norovirus survives under dry conditions and in liquids and causes new cases of infection through long-term transmission routes without directly infected individuals. If sewage treatment is insufficient, norovirus in human and livestock feces migrates into the sea through rivers, particularly after heavy rains. Norovirus was subsequently accumulated and concentrated in bivalves such as oysters. Consumptions of contaminated raw oysters cause new infections. When food manufacturers or workers in the food industry are infected with norovirus, food-derived viral transmission may occur among many people and may spread rapidly.

Prevention of viral transmission is the most effective measure to reduce norovirus infections because of the lack of a specific vaccine or drug, as well as the practical complexity associated with rapid virus-testing by the food inspection agency. In particular, education and training on risk management of norovirus is an extremely important requirement even at the final stages of the food production process chain due to the occurrence of the transition from home-cooking to eating-out and/or takeaway meals.

Risk communication is based on information transfer directly from the government to the consumer. The direct dispersion to consumers requires greater human resource involvement. Therefore, we have investigated the possibility of dissemination of government’s food safety information through opinion-leaders. Food Safety Commission of Japan (FSCJ) conducted a questionnaire survey among their monitors to determine their level of knowledge of risk management with regard to oysters for raw consumption and norovirus. This study investigated the level of knowledge of risk management with regard to oysters for raw consumption among people with an interest in food, and intended to obtain appropriate measures for risk communication to prevent food poisoning associated with raw oyster consumption.

## 2. Materials and Methods

Total 405 persons qualified from the FSCJ monitors in 2018 participated in the present study. The respondents were 348 persons (85.9%). Judged from anonymised data of the FSCJ, 27.3% of the study participants were associated with food production, 15.5% in the food retail business, 11.8% in food research, 9.2% in medical care, 12.9% in education, 6.6% in food administrative officer, and 16.7% in other occupations as their past or present jobs.

A questionnaire generated in the FSCJ was delivered to the monitors to reply the following questions through the Web-input system “Nopi” in 2018.

Question 1: State whether the answers to each of the six questions about norovirus as a health hazard in Table 1 are true or false.

**Table 1.** Questions with regard to norovirus as a health hazard and responses from monitors (n = 348). All questions of "a-f" are correct answer.

Questions	Ratio of respondents with correct answers (%)
a. Oysters contaminated with norovirus can be inactivated by heating at 85–90°C for more than 90 s	72
b. Food poisoning cases of norovirus caused by transmission from a food worker are higher than the number of cases caused by consumption of oysters	75
c. Dried foods, such as chopped seaweed, can also cause norovirus food poisoning	80
d. Norovirus in contaminated oysters is not inactivated by freezing	80
e. Sodium hypochlorite should be used to dispose the vomitus of patients with norovirus food poisoning	89
f. Handwashing is effective in preventing norovirus infection, but washing with soap for 10 s and then rinsing with running water for 15 s is not enough	78
Ratio (%) of respondents with correct answers for all questions	45
Mean score	79

Question 2: State whether the answers to each of the six questions on current risk management for oysters for raw consumption in Table 2 are true or false.

**Table 2.** Questions on current risk management for oysters for raw consumption and responses from monitors (n = 348). All questions of "a-f" are correct answer.

Questions	Ratio of respondents with correct answers (%)
a. Microbiological standards of oysters for raw consumption are determined by the most probable number of *E. coli*, the most probable number of *Vibrio parahaemolyticus*, and the number of bacteria	65
b. Processing standards of oysters for raw consumption do not check for norovirus	47
c. Processing standards of oysters for raw consumption do not specify sea area standards	65
d. Water used for peeling the meat of oysters must be potable water, sterilized sea water, or artificial sea water created by using potable water	68
e. The ingredient and processing standards of oysters for raw consumption are specified by the Ministry of Health, Labour and Welfare	58
f. Oysters for raw consumption must be labeled “for raw consumption”	83
Ratio (%) of respondents with correct answers for all questions	27
Mean score	64

After two weeks, the FSCJ have collected the questionnaires from monitors and counted the answers.

## 3. Results and Discussion

Tables 1 and 2 are the summary of results from Questions 1 (Q1) and 2 (Q2) provided by the FSCJ monitors. For Q1, 45% of the participants selected the correct answers for all questions. The mean score reached to 79%. These values were considerably higher than the 27% and 64%, respectively, for Q2. The questions of norovirus were resulted in the high rates of correct answers (72–89% for Q1a–f, and 83% for Q2f). On the other hand, the rates of correct answers remain relatively low (47-68%) to Q2a–d, which were specific questions on risk management of oysters for raw consumption. These correct answer rates were lower than those for norovirus. The rate of correct answers for Q2b for the inspection subject was 47%.

Monitors with job experience in the food industry had high levels of knowledge on how to prevent infection from norovirus with regard to Q1. They might be not enough for knowledges of risk management on the preparation for oyster low-consumption as ascertained with Q2 as a cause of food poisoning. As described in the reply rate for Q2a, more than half of the monitors misunderstood the microbiological standards for oysters for raw consumption. The standard was based on the bacteria counts of *Escherichia coli *(*E. coli*) and *Vibrio parahaemolyticus*^5^^)^, and not the count of norovirus (Q2b) causing food poisoning in humans. As noted in Q2c, the processing standards for oysters for raw consumption is the “sea area standards” for cultivating oysters. However, many monitors may have been misled by the term “standards” because of being unaware that the standard that was being referred to here was the *E. coli* count in seawater.

The detection of *E. coli*, which is a commensal in the intestinal tract of humans or livestock, indicates the possibility of fecal contamination^2^^,^^3^^,^^4^^)^. The *E. coli* count in raw oysters and seawater are regarded as indicators of fecal contamination and are set to legal standards universally. In other countries as well, the risk management of oysters involves emphasis on the proper treatment of sewage and the maintenance of clean sea areas. In the European Union, the management rules for oysters include control of sewage pollution, classification of aquaculture areas by *E. coli* counts, processing of oysters for sale, and quality control of the food products^6^^,^^7^^,^^8^^)^. 

Consumers in countries do not recognize that the primary mode of transmission of norovirus infection is orofecal^9^^,^^10^^)^. Risk communication still have a room for debate whether the public should be informed about the accumulation of norovirus in oysters which partially stems from an insufficient fecal decontamination during the treatment of sewage. Moreover, this issue linked to difficulty in interpretation since the current risk management item is not based on the presence of norovirus but is based on the *E. coli* count. 

The results of a one-way analysis of variance between the score for Q2 on risk management of oyster raw-consumption and extent of the monitor’s job experiences are shown in Table 3. The averages of all questions in Q2 were unequal among the groups constituted by the monitor’s job experience. The high score for a food-related government employee is not unexpected since they are risk management experts. Researchers are generally in superior positions in the instruction system for quality assurance and labeling among food companies; therefore, monitors with research experiences also had high scores. Monitors with experiences in food production or retailing were specialized in their area of responsibility and were the third only to the researchers in terms of the questionnaire responses. The low scores of Q2 for medical workers and educators are probably due to few opportunities to learn the practical risk management of oysters for raw consumption, although they have the knowledge of noroviruses owing to the high scores of Q1. Therefore, the lowest point Q2b, which is specific management information among Q2, was influenced by the occupational experience of monitors. The factors other than the monitor’s work experience, such as sex, age, frequency of eating raw oysters, and experience of food poisoning by eating raw oysters did not contribute to the correct answer rate in Q1 and Q2, using cluster analysis (data not shown).

**Table 3.** Results of one-way analysis of variance between the score for Question 2 and the population with monitor work experiences (n = 348).

Monitor’s job experience	**n**	Average	
		All questions a-f	Question b
Production or processing for food	95	3.96	0.42
Retail or sales for food	56	4.13	0.52
Research for food	41	4.44	0.63
Administrative official for food	23	4.48	0.65
Medical or education	77	3.49	0.44
Others	58	3.31	0.38
Observed dispersion ratio		3.67	2.24
*F*-critical value		2.24	2.24
*P*-value		0.003	0.050

The questions conducted simultaneously on the monitors indicated food poisoning as the top of their most anxious and concern and also as their high risk-perception for food poisoning^11^^)^. In order to further permeate the risk management of oyster raw-consumption to opinion-leaders such as monitors, information with precise terms and explanations is encouraged. The way and device of communication for hygiene management needs to be improved and precise terms. The current management system^5^^)^ has been instituted since 1967, and government and fisheries organizations continued to deliver information through documents and web pages. However, this system is not easy for cooks and consumers to understand. The results of the present study, together with a FSCJ-conducted survey^12^^)^ towards general consumers in 2007, indicate that current knowledge is not appropriate among consumers and opinion-leaders for food hygiene. In the area of research and development, the improvements of analytical technology, which allow rapid extraction, selective detection of active noroviruses and polymerase chain reaction with detection suppression of inactive noroviruses, are being promoted, in order to standardize the number of active noroviruses in oysters^4^^)^. High-pressure treatment technologies to inactivate norovirus in raw-oysters is planned to become commercialized^4^^)^. However, until these innovations are implemented, the dissemination of precise information is needed with regard to the current hygiene system to all individuals in the supply chain – from oyster producers to the final consumers. The effective measures for risk management of oyster for raw consumption include, 1) the government disseminates the information for oyster hygiene management to social leaders in an easy-to-understand contents, and 2) they encourage the dissemination of the information to cooks and final consumers.
